# Unified Environment for Real Time Control of Hybrid Energy System Using Digital Twin and IoT Approach

**DOI:** 10.3390/s23125646

**Published:** 2023-06-16

**Authors:** Lamine Chalal, Allal Saadane, Ahmed Rachid

**Affiliations:** 1 Icam School of Engineering, Lille Campus, 6 Rue Auber, B.P 10079, CEDEX, 59016 Lille, France; allal.saadane@icam.fr; 2 Laboratory of Innovative Technologies, University of Picardie Jules Verne, 80000 Amiens, France; rachid@u-picardie.fr

**Keywords:** digital twin, internet of things, real-time monitoring, rule-based control, battery, standalone photovoltaic system

## Abstract

Today, climate change combined with the energy crisis is accelerating the worldwide adoption of renewable energies through incentive policies. However, due to their intermittent and unpredictable behavior, renewable energy sources need EMS (energy management systems) as well as storage infrastructure. In addition, their complexity requires the implementation of software and hardware means for data acquisition and optimization. The technologies used in these systems are constantly evolving but their current maturity level already makes it possible to design innovative approaches and tools for the operation of renewable energy systems. This work focuses on the use of Internet of Things (IoT) and Digital Twin (DT) technologies for standalone photovoltaic systems. Based on Energetic Macroscopic Representation (EMR) formalism and the Digital Twin (DT) paradigm, we propose a framework to improve energy management in real time. In this article, the digital twin is defined as the combination of the physical system and its digital model, communicating data bi-directionally. Additionally, the digital replica and IoT devices are coupled via MATLAB Simulink as a unified software environment. Experimental tests are carried out to validate the efficiency of the digital twin developed for an autonomous photovoltaic system demonstrator.

## 1. Introduction

Renewable energy systems, such as solar panels and wind turbines generate power from natural resources that are available intermittently. Since they produce variable power, their effective dissemination can be accelerated by better control and monitoring. Traditionally, data acquisition systems (DAQs), usually centralized, are used for collecting all system data [1]. However, the cost of commercial DAQs is the most significant barrier for greater diffusion. IoT (Internet of things) based smart meters have recently gained substantial popularity for control and measurement data. Indeed, their ability to communicate data over networks offers a wide range of applications. Therefore, IoT devices can potentially be useful for real-time energy management. Accordingly, energy production efficiency and reliability can be significantly improved ([2,3,4]). This can help reduce dependence on traditional fossil fuel-based energy sources and promote renewable energy.

However, implementing IoT in renewable energy systems faces several challenges due to the variety of protocols and devices available in the market [5]. This can make it difficult to integrate IoT devices while ensuring their compatibility with the existing infrastructure [6].

On the other hand, defined as the combination of the physical system and its digital model, the Digital Twin (DT) paradigm can be used to predict energy production and consumption. It also enables predictive maintenance [7]. Therefore, it is crucial to implement techniques that provide a wide range of operational data about the actual system [8]. IoT technology can be associated with DT thanks to their ability of actuation and sensing. 

**Figure 1:**
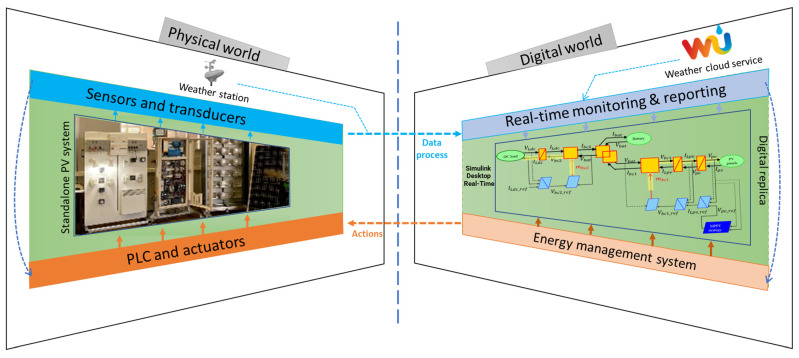
Diagram of the digital twin of the standalone PV system.

In this work, our objective is to build a DT of a standalone PV system to deal with real-time energy management challenges. Figure 1 depicts the diagram of the digital twin as developed in this work:

- Physical system: made up of two PV panels, batteries, DC loads, a solar emulator, and a weather station.
- Smart sensors and actuators: including devices required for control and monitoring purposes.
- Digital counterpart implemented in MATLAB: including mainly EMR-based model, Real-Time monitoring, and control systems.

To experimentally validate the proposed framework, we have developed a lab demonstrator according to IoT-based architecture for embedded and distributed instrumentation. Furthermore, to cope with the devices’ heterogeneity, we use MATLAB Simulink. Indeed, this is a comprehensive software environment that can communicate with sensors and Programmable Logic Controllers through client/server applications.

This document is structured as follows. First, we present a review of the literature related to our work. In the second part, we detail our test bench which constitutes the physical part of the proposed Digital Twin. In the third part, we present an approach based on the Energetic Macroscopic Representation (EMR) formalism as a numerical counterpart of our system. Finally, the experimental validation of the resulting digital twin will be discussed.

## 2. Literature Review

Several studies have considered photovoltaic systems monitoring. In [9], the authors used the IoT and MQTT (MQTT: Message Queuing Telemetry Transport) in web-based monitoring. They implemented this approach to monitor the performance of the solar panels and the battery system, as well as the energy consumption of a living laboratory. Similar work is reported in [10]. When compared to other protocols, MQTT has a small footprint, making it much more suitable for resource-constrained environments. Despite several benefits, it is important to note that MQTT brokers do not provide the same level of entity authentication or encryption capabilities [11]. Moreover, IoT devices are often not interoperable, and it is difficult to integrate external sources of information and cloud computing to use energy more efficiently. Indeed, it requires the design and implementation of hierarchical architectures and standardized solutions to facilitate interoperability. Till today, no standard solution is established yet [12]. However, most providers share IoT middlewares, which has fostered the emergence of cross-domain applications.

In [13], the authors developed a Smart Home monitoring system using Power Line Communication (PLC) which has the advantage of not needing additional communication cables [14]. This article also demonstrates the potential of using PLCs to monitor individual photovoltaic panels.

Moreover, the review work [1] provides an overview on the importance of monitoring systems for photovoltaic plants (electrical and meteorological data). The article reviews different types of monitoring systems that are currently available for PV plants, including hardware and software aspects. The authors discuss the advantages and disadvantages of each type of system and provide examples of commonly used components. 

There exist several commercial software for monitoring and simulation of PV systems such as LabVIEW ([15,16,17,18]) and MATLAB Simulink ([19,20]). 

Furthermore, studies in [20,21,22,23,24], present an energy management system by using Programmable Logic Controller. Compared to other hardware control systems, PLCs have specific advantages as ruggedness, noise immunity, modularity, low cost, and small footprints [25]. 

We also reviewed several papers dealing with Digital Twins. This concept is particularly popular in the context of industry 4.0, where it is mainly implemented for manufacturing systems [26]. Nowadays, there is a significant trend to apply this concept to the electrical energy field [27]. That said, there are few concrete applications. Moreover, as there are several misconceptions about digital twin definition [28], it is important to distinguish the digital twin, the digital model, and the digital shadow. In fact, the digital model is defined as a digital copy of a physical system without any data exchange and is generally used for simulation and design purposes. Likewise, the Digital Shadow is a combination of a physical system and its digital model with a one-way data exchange.

Although some authors claim to use the concept of the Digital Twin as just defined above, most of the articles deal with the “digital model” or “digital shadow”. For instance, authors in [27] study the digital twin possibilities for fault diagnosis purpose of PV system. However, they use a digital shadow instead of digital twin. We find the same confusion in the articles [29,30,31,32,33].

Relatively to our contribution, Table 1 summarizes the state of art of the current literature dealing with digital twin applications.

In this work, we propose a Digital Twin of a complete photovoltaic system using MATLAB software as a unified environment. This framework is suitable to address the following topics:

- Real-time access to multi-protocol data for monitoring purposes.
- Modelling, simulation, and real-time control of PV systems
- Implementing of innovative energy management systems
- Reporting

In other words, this experimental platform can be used to compare simulation results and monitored data in real time. Indeed, it could be used to develop new approaches for fault detection and prediction issues. This integrated environment allows on the one hand to have a large panel of toolboxes (modelling, code generation, machine learning, advanced control, cloud computing…). Therefore, it could be easily used for advanced control and optimization purposes.

## 3. Materials and Methods

The concept of the digital twin, object of this work, is implemented on a test rig. In this section we describe the structure of the demonstrator, its instrumentation and control system.

### 3.1. PV System Description

**Figure 2:**
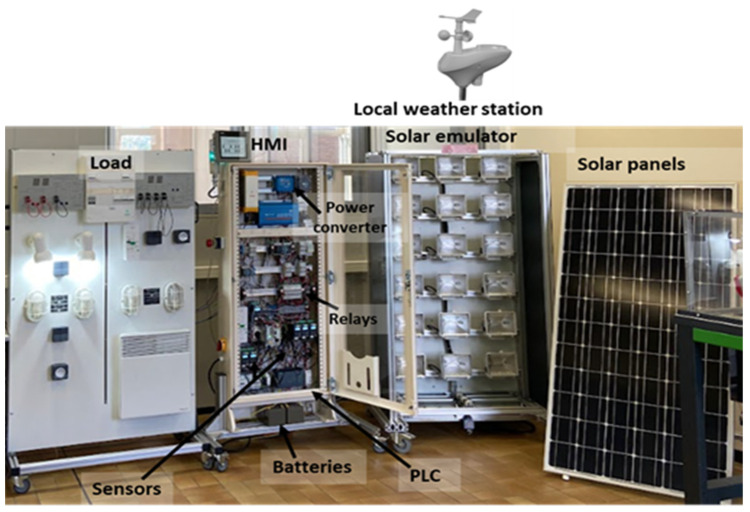
PV system demonstrator.

The stand-alone system is composed of the following elements (Figure 2):

- Solar emulator as artificial light source,
- 2 × 215 Wp photovoltaic panels (SunPower Co., San José, CA, USA),
- 28 Ah batteries as storage system (Victron Energy B.V., Almere, The Netherlands),
- DC loads.
- Power converters (Victron Energy B.V., Almere, The Netherlands).

### 3.2. Sensors and Data Aquisition

Developing a digital twin for renewable energy systems requires constant data collection and monitoring [40]. Therefore, it is necessary to implement techniques that provide a wide range of data:

- Weather data (temperature, irradiance, wind speed…);
- Real time electrical data (energy, currents, voltages, batteries’ state of charge…).

The experimental platform is equipped with sensors of various technologies that do not use the same communication protocol. To transmit measurement data to a unified software environment, we have developed a hardware and software architecture based on the following components:

- An IoT architecture combining smart sensors for electrical data [41] and a weather station. As IoT, these devices use heterogeneous communication protocols [42], including Modbus TCP/IP, HTTP, and PROFINET;
- MATLAB Simulink, as an integrated environment concentrating all the operational data of the system, constitutes the core of the digital twin.

**Figure 3:**
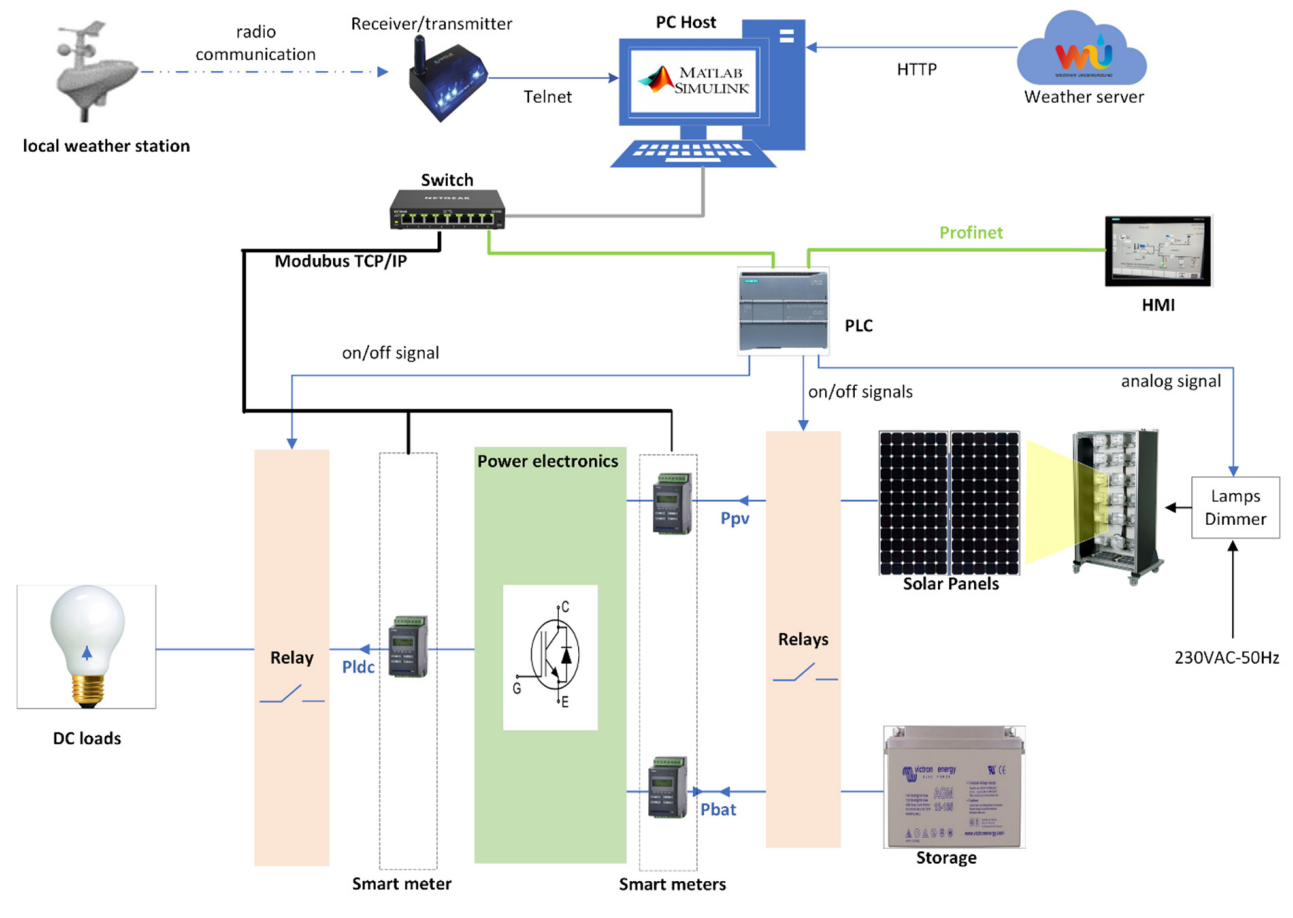
Control system architecture of the PV plant.

Figure 3 depicts the demonstrator overview and its control system architecture.

### 3.3. Control System

At first level, the embedded control system is based on a S7-1200 PLC which controls solar emulator and relays. This allows experimental tests to be carried out according to real-time weather data or with historical data.

At the second level, the overall control of the PV system is performed within MATLAB Simulink programs using the embedded PLC as a slave. Data communication is carried out using OPC-UA and Industrial Communication Toolbox™.

**Figure 4:**
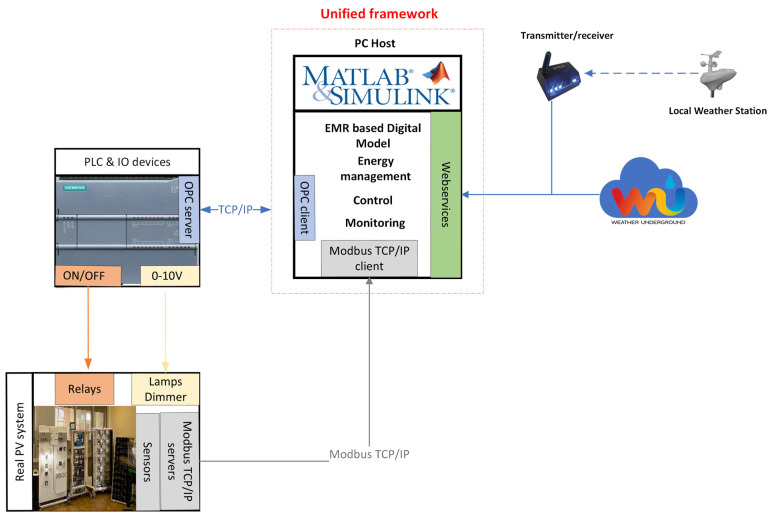
Digital Twin architecture overview.

Figure 4 describes the unified architecture proposed in this work.

## 4. PV System Modeling

This section details our approach to modeling the PV system. The aim is to develop the digital replica counterpart of the Digital Twin.

### 4.1. Solar Panels

A photovoltaic (PV) power system consists of two solar panels (SPR-215-WHT). PV parameters of each panel are given in Table 2.

**Figure 5:**
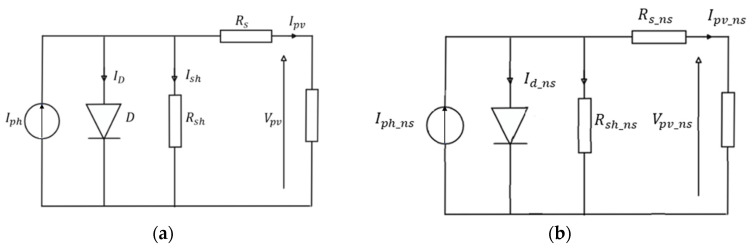
Equivalent circuit model. (**a**) PV cell; (**b**) PV panel composed of $n_{s}$ cells in series.

PV panel is composed of several cells. Each PV cell is made up of semiconductor materials which can convert solar irradiance into electrical energy. Based on the electronics theory of semiconductor p-n junction, it can be described by a current source. The studied panel model in this work is represented by an equivalent circuit. It consists of a single diode for the cell polarization function and two resistors (series and shunt) for the losses (Figure 5). The equivalent circuit is composed of an ideal current source $I_{ph}$ in parallel, reverse diode, series resistance $R_{s}$ and parallel resistance $R_{sh}$.

The $I_{pv}=f\left(V_{pv}\right)$ characteristic of this model is given by the following equation:

(1) $$I_{pv}=I_{ph}-I_{D}-\left(\frac{V_{pv}+R_{s}I_{pv}}{R_{sh}}\right)$$

where:

- $I_{pv}$ is the current generated by solar panel;
- $I_{ph}$ is the photocurrent, which is linearly proportional to irradiance and depends on the temperature as shown in the following equation:
  $$I_{ph}={(I}_{phn}+\alpha_{sc}\Delta T)\frac{G}{G_{n}}$$
  ○ where $\Delta T={T-T}_{n}$ ($T_{n}=25{}^{\circ}C$), G is the incident of irradiation on the solar panel, and $G_{n}$ (1000 W/m²) at standard conditions (STC);
- $I_{D}$ is the diode current:
  (2) $$I_{D}=I_{0}[\exp\left(\frac{qV_{pv}}{AKT}\right)-1]$$
  ○ where: $I_{0}$ is the PV cell reverse saturation current that mainly depends on the temperature, q is the electronic charge of an electron ($1.6\times 10^{19}$ C), *T* is the temperature of the PV cell, *k* is Boltzmann’s constant ($1.38\times 10^{23}$ J/K), and A is the diode ideality factor.

A PV panel is made up of numerous identical PV cells connected in series to provide a higher voltage. A PV module composed of $n_{s}$ identical cells in series can also be represented by the equivalent circuit shown Figure 4a but the circuit needs to be modified [43] as follows (Figure 5b):

(3) $$R_{s\_ns}=n_{s}R_{s},\;R_{sh\_ns}=n_{s}R_{sh},\;A_{ns}=n_{s}n,\;I_{0\_ns}=I_{0},\;I_{ph\_ns}=I_{ph}$$

The solar panel datasheet does not include some important parameters, such as $R_{s}$ and $R_{sh}$. To obtain them, we used the PV array tool of Matlab by setting the PV parameters of the studied solar panel. Table 3 shows the extracted parameters.

**Figure 6:**
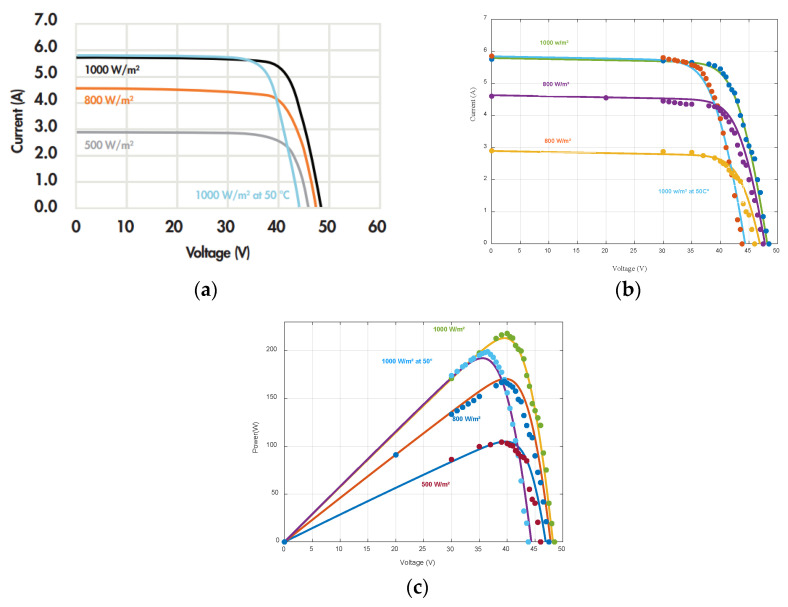
Actual data vs. data computed by the model: (**a**) I-V characteristics from datasheet; (**b**) actual I-V characteristics vs. computed I-V characteristics; (**c**) actual P-V characteristics vs. computed P-V characteristics.

The mathematical model of the studied solar panel is then implemented under MATLAB Simulink and the results are compared to datasheet data as shown in Figure 6.

The dotted curves in Figure 5 represent data from the datasheet and the continuous ones come from the mathematical model. These results show a good correlation between the model and the solar panel manufacturer’s data.

**Figure 7:**
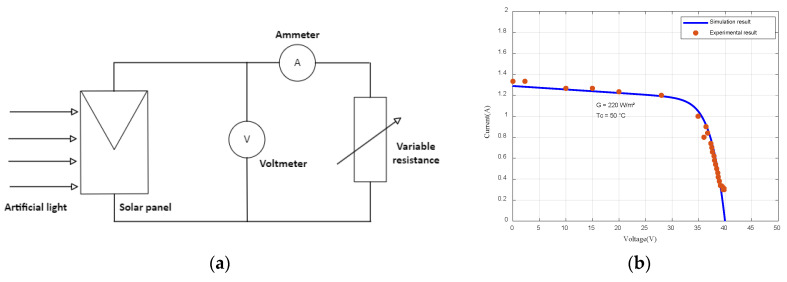
Experimental test and comparison of I-V curves: (**a**) experimental components of the tested PV panel; (**b**) comparison between real test and the mathematical model.

To assess the model’s effectiveness, we conducted indoor tests of the PV panel. The PV panel was illuminated using an artificial light source, while its output was connected to a rheostat. To obtain the I-V curves, measuring instruments were integrated, as depicted in Figure 7a.

Figure 7b shows the simulation and experimental results, confirming the accuracy of the implemented mathematical model.

### 4.2. Batteries Bank

Due to the inherent variability of PV power, the battery plays a crucial role in stand-alone PV systems. In this system, two 12 V AGM batteries are installed and connected in a series configuration. The utilization of AGM batteries provides additional benefits, as they facilitate recombination and effectively mitigate gas emissions during overcharge. Consequently, the demand for room ventilation is reduced, and the batteries do not emit any acid fumes during normal discharge operations [44].

**Figure 8:**
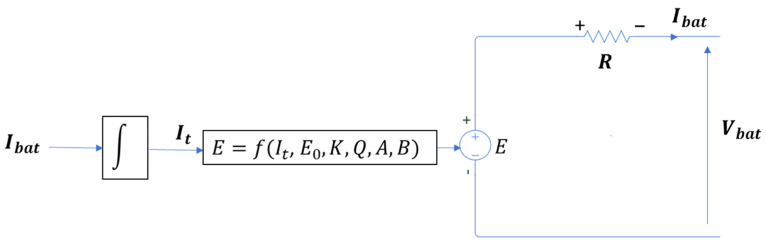
Nonlinear Battery model.

The battery model in Figure 8 considers a constant internal resistance. This resistance is connected in series with the internal voltage source which depends on various parameters [45].

The terminal voltage of the battery is given by:

(4) $$V_{bat}=E-RI_{bat}(t)$$

where:

- R: is the internal resistance;
- $I_{bat}$: is the battery current.

The controlled voltage source E is described by the following equation:

(5) $$E=E_{0}-K\frac{Q}{Q-\int I_{bat}(t)dt}+Aexp(-B\int I_{bat}(t)dt)$$

where:

- $E_{0}:$ Fully charged voltage;
- K: polarization voltage (V);
- Q: battery capacity (Ah);
- A: exponential zone amplitude (V);
- B: exponential zone time constant inverse (Ah).

The parameters of this equivalent circuit can be identified by considering the discharge characteristics with a nominal current.

We use a lead-acid battery, which features a nominal voltage of 12 volts (*E*_0_), a capacity of 14 Ah (Q), a rated current of 0.7 A, and an internal resistance of 14 mΩ (Table 4).

**Figure 9:**
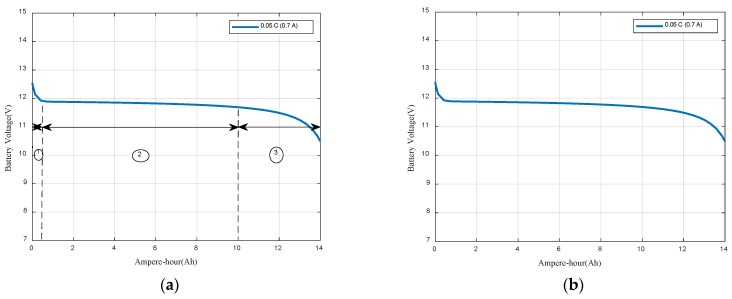
(**a**) Discharge curve (Q-V); (**b**) Discharge curve (Hours-V).

The model parameters (K = 0.12, A = 0.66, B = 7) are obtained through graphical estimation based on the rated current discharge characteristic curve. The simulation results are depicted in Figure 9. The Q-V discharge curve is composed of three sections. The first section represents the exponential voltage drop if the battery is initially fully charged. The second phase represents the charge that can be extracted from the battery until the voltage drops below the battery nominal voltage. Finally, the third section represents the total discharge of the battery, when the voltage drops quickly. The width of these sections depends on the battery type.

To estimate the state of charge of the battery (SoC), the well-known Coulomb counting method is used due to its simplicity. It relies on measuring the current and the estimation of the initial state of charge of the battery.

(6) $$SoC\left(t\right)={SoC}_{0}-\frac{1}{C_{nom}}\int_{t_{0}}^{t}i_{batt}\left(t\right)dt*100$$

### 4.3. Power Electronics

**Figure 10:**
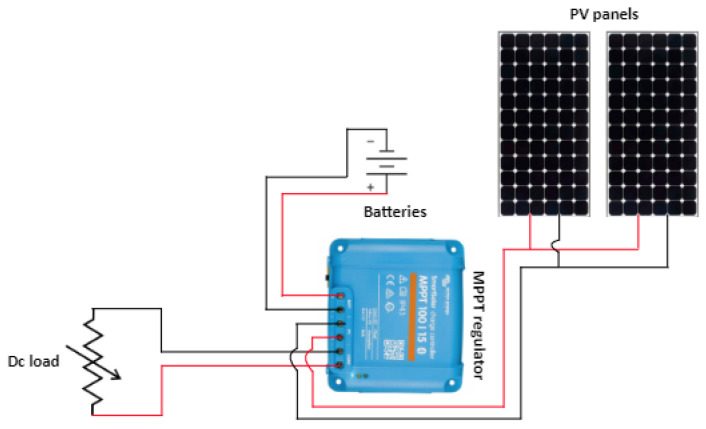
Simplified PV system architecture.

Figure 10 illustrates the simplified PV system architecture, comprising a solar panel, a battery for energy backup, and power converters (MPPT regulator) that connect these components to the DC load.

**Figure 11:**
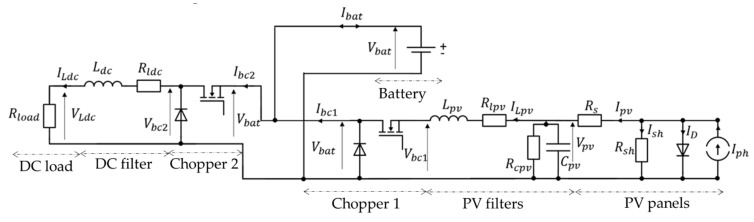
Topology of stand-alone hybrid PV system.

We determined the converter topology based on the current state of the art since it was not specified by the manufacturer. Photovoltaic energy harvesting relies primarily on irradiance and solar panel temperature, resulting in variable PV voltage. Therefore, implementing Maximum Power Point Tracking (MPPT) is essential to maximize power extraction regardless of weather conditions. A DC-DC converter is used to enable voltage adjustment from the photovoltaic panels to match the battery voltage. This converter performs step-up and step-down functions by controlling the input-to-output voltage ratio through the variation of the converter switching device’s on-off duty cycle. Another converter is required to supply and regulate the voltage for the DC load. In the literature, this configuration is commonly referred to as a two-stage DC-DC converter (Figure 11), as reported in [46].

### 4.4. EMR Representation of the Digital Replica

In the proposed system, the battery imposes a constant voltage, and the PVs relates to the battery through the buck converter. It enables effective Maximum Power Point Tracking (MPPT) control for the PV panels. Moreover, RC ($R_{cpv},C_{pv}$) and RL ($R_{lpv},L_{pv}$) filters are inserted between the PV and the buck converter to reduce voltage and current ripple. Finally, the battery is connected with the DC Load via a chopper and a RL ($R_{ldc},L_{dc}$) filter.

EMR (Energetic Macroscopic Representation) is a graphical formalism for organizing models and control of subsystems within a complete system [47]. The advantage of the EMR formalism lies in its ability to provide a comprehensive and systematic approach for modeling and analyzing complex energy systems. Firstly, each component is translated into EMR elements, and their inputs and outputs are defined according to the causality principle (action/reaction). Moreover, the system is decomposed into basic subsystems with interactions using colored pictograms (orange and green). Furthermore, the control blocks are depicted as blue parallelograms. Table 5 depicts the main EMR elements.

**Figure 12:**
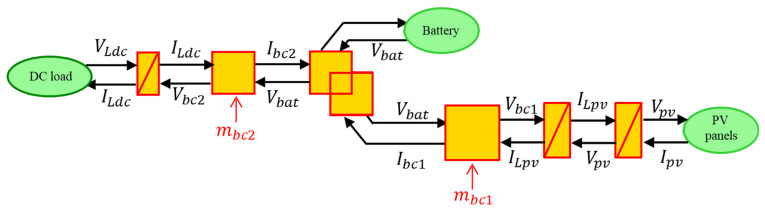
EMR of the studied hybrid system.

Based on the information on all parts of the demonstrator and hypothesis, the EMR (Energetic Macroscopic Representation) of the studied system is designed. The modeling methodology and the EMR organization method are provided in [49]. In Figure 12, the entire EMR organization of the Digital model is depicted.

**Figure 13:**
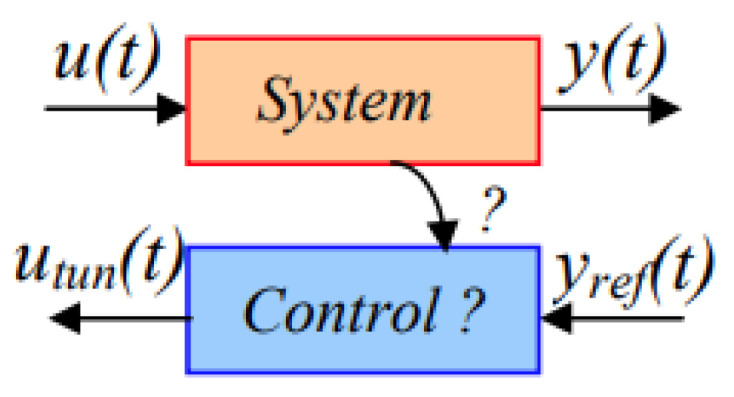
Inversion-based control principle.

The control chains are deduced using inversion control theory [50] where the control structure of a system is considered as an inverse model of the system (Figure 13).

**Figure 14:**
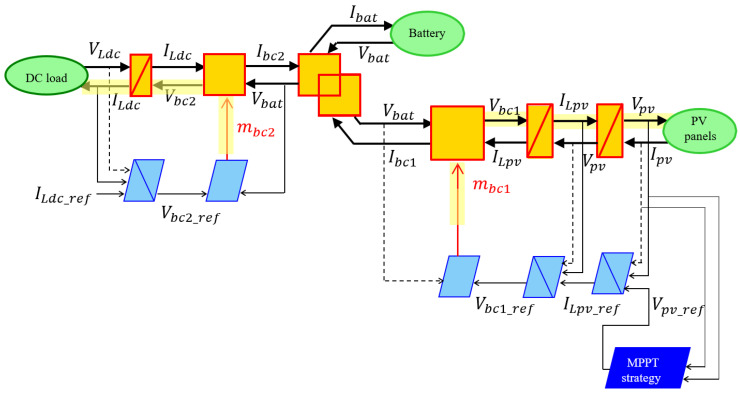
EMR and deduced control of the studied hybrid system.

The tuning paths (yellow lines in Figure 14) are defined according to the following objectives:

1. Extract the maximum of the solar power by acting on chopper 1 to find the optimal solar panels voltage;
2. Satisfy the DC load demand by acting on chopper 2.

The control scheme of the hybrid system is obtained by inverting the EMR element by element according to the tuning chains (see lower part of Figure 14).

Conversion elements are inverted directly as they have no time-dependence behavior. The accumulation elements (rectangle with forward slash) cannot be inverted physically to avoid derivation. Thus, an indirect inversion is made by using IP controller. Table 6 shows three examples of direct and indirect inversion.

**Figure 15:**
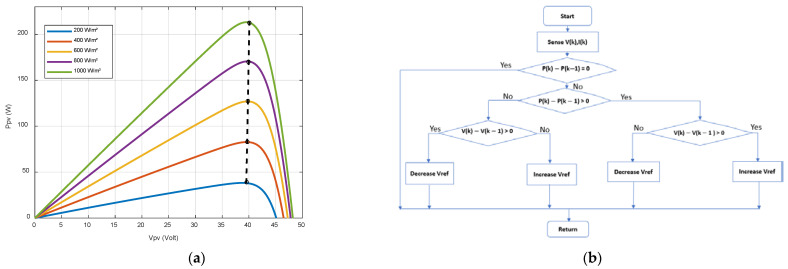
(**a**) PV Power versus solar panel voltage for different irradiance (T = 25°) (**b**) Flowchart of perturb and observe algorithm.

The MPPT (Maximum Power Point Tracking) algorithm is widely used in PV systems. In this study, the Perturb and Observe (P&O) method is implemented. As shown in Figure 15a, the power curves versus the PV panels output voltage present maximum power points (empty circles). A Perturb and Observe Maximum Power Point Tracking strategy [51] is implemented to define the reference voltage imposed on PV panels to obtain the maximal PV power whatever the irradiance and temperature are (Figure 15b). The P&O algorithm begins by sensing PV voltage and current voltage. The value of the current power (V(k) X I(k)) is then compared to the previous power measurements. If the difference between the two measurements is equal to zero, then the value of the voltage is used as a reference to control the PV voltage thanks to the chopper. If the value of the difference is not equal to zero and if an increase in PV voltage generates an increase in power, this means that there is a convergence to MPP (Maximum Power Point). However, if the power decreases, the PV voltage reference must be reduced to converge to the MPP. The developed algorithm is implemented by the MPPT strategy block (dark blue block) as shown in Figure 15a.

**Figure 16:**
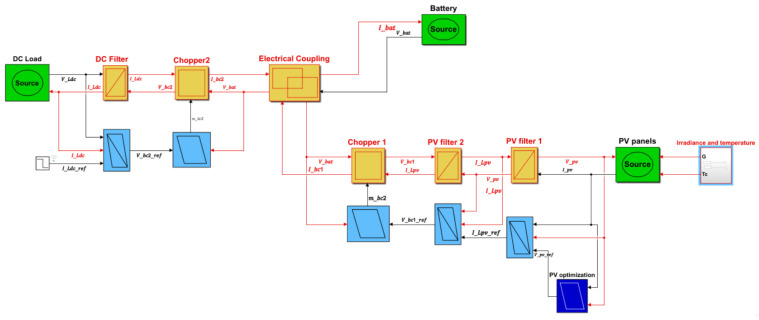
MATLAB Simulink model of the studied hybrid system and its control.

The following figure (Figure 16) presents the implemented digital model under MATLAB Simulink thanks to a Simulink library containing the EMR basic pictograms.

**Figure 17:**
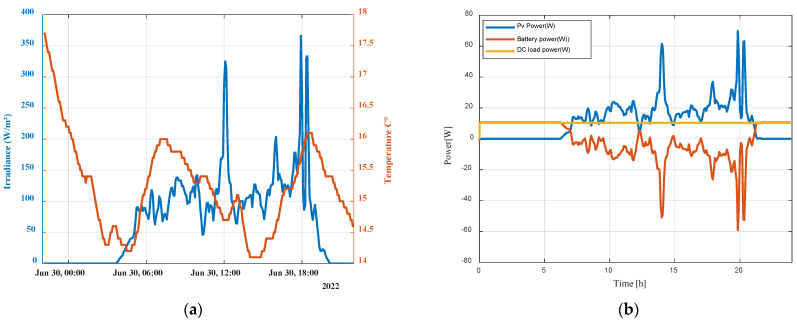
(**a**) Monitored irradiance and temperature profiles; (**b**) PV, battery, and load power curves.

To evaluate the effectiveness of the developed digital model, simulations have been carried out with real-time solar irradiance and temperature data (Figure 17a) for a typical day (30 June 2022). The digital model can be run and connected to real time weather conditions thanks to Simulink Desktop Real Time. This latter provides a real-time kernel for executing Simulink models on a laptop or desktop running Windows or Mac OS X.

Figure 17b shows simulation results of $P_{pv}$, $P_{bat}$, $P_{DCl}$. PV panels cannot provide enough energy to feed the DC load ($P_{pv}$ < $P_{DCl}$ = 10 W) before the time 0.1 s, 6:30 a.m. Thus, the battery provides the DC load during this period. After 6:30 a.m., the photovoltaic panels begin to produce electricity and the battery provides the difference to satisfy the battery. When PV power exceeds the load demand, the battery is charging.

**Figure 18:**
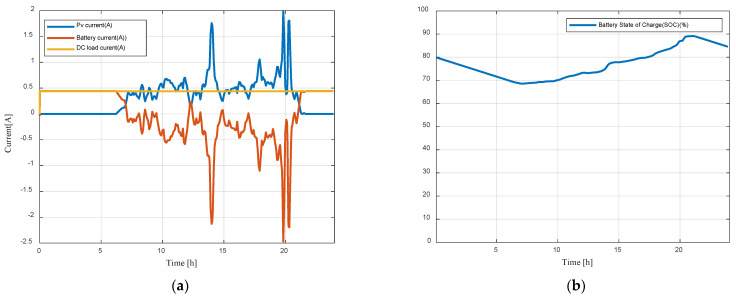
(**a**) PV, Battery, and DC load currents; (**b**) Battery state of charge.

Figure 18a shows simulation results for $I_{pv}$, $I_{bat}$, $I_{DCl}$ while Figure 18b shows the state of charge of the battery. The battery is discharging when PV panels output is insufficient, and it is charging when PV power is higher than DC load demand.

## 5. Experimental Results and Discussion

Real-time measurements were carried out to show the effectiveness of the proposed unified framework, where the three electromagnetic relays are activated. These measurements are subsequently compared in real time with the digital model data, as outlined in Figure 1.

**Figure 19:**
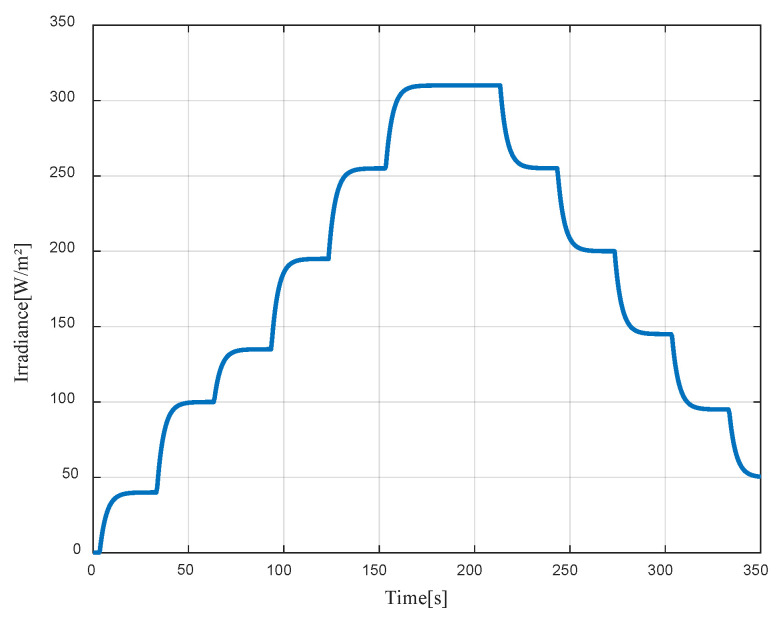
Applied solar irradiance (artificial light source).

The applied solar irradiance is presented in Figure 19. From the figure, we can see that the artificial source causes a slight delay and behaves like a first-order system with non-linear behavior depending on the increase or decrease in light. This is due to the light dimmer.

**Figure 20:**
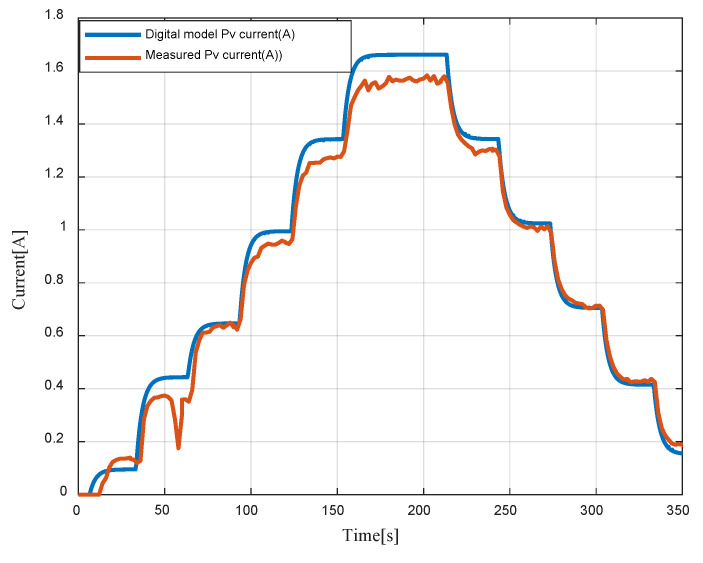
Digital model PV current vs measured PV current.

Figure 20 represents the measured PV current and the PV numerical model, highlighting a slight deviation observed in the illumination levels around 300 W/m². This difference is due to both the approximation of the mathematical model and the non-uniform illumination of the PV panels. Furthermore, it should be noted that the maximum relative error is approximately 6%, and the maximum absolute error is around 0.1, which is considered low.

**Figure 21:**
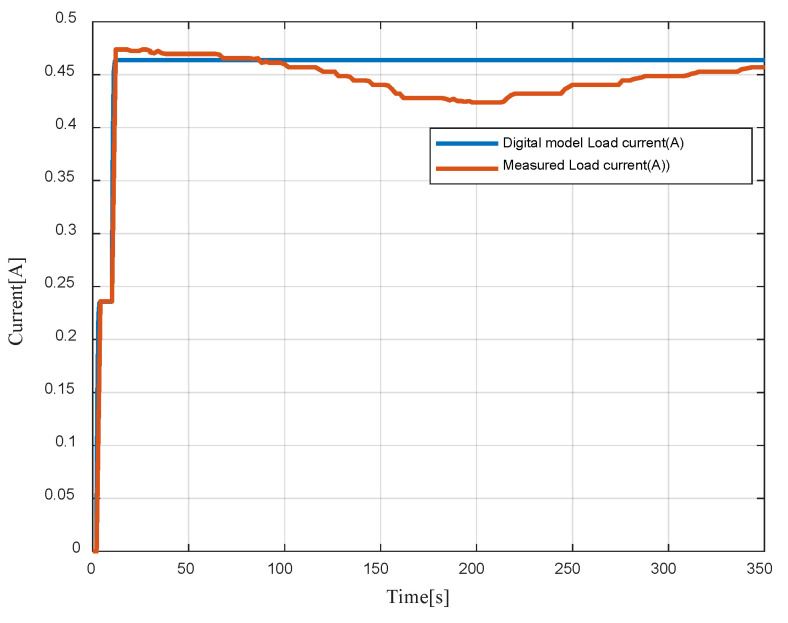
Digital model vs measured load currents.

In Figure 21, the measured and digital model load currents are depicted. The tests were initiated by activating a single lamp and subsequently both lamps. This led to an increase in the DC current from 0.23 A to 0.46 A.

**Figure 22:**
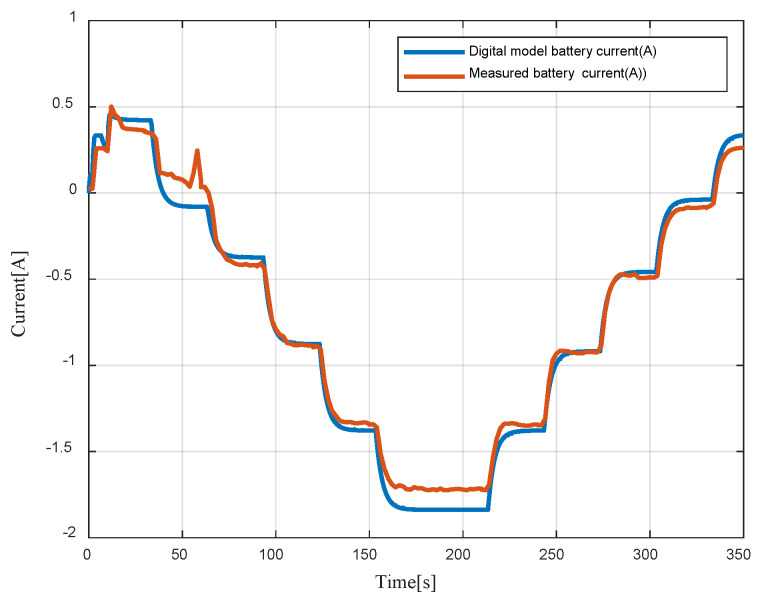
Digital model battery current vs measured battery current.

Measured and digital model battery currents are depicted in Figure 22. A positive current refers to a battery discharge, while a negative current refers to the charging phase.

**Figure 23:**
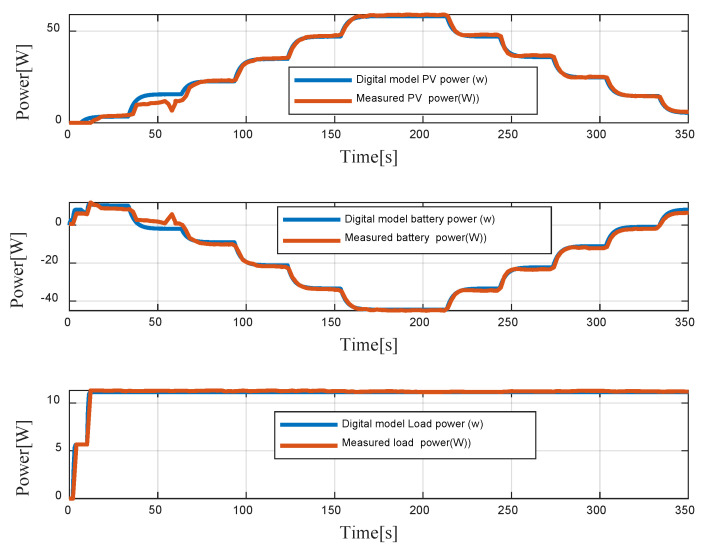
Digital model data vs measured PV, battery, and load power.

Figure 23 represents PV power, battery power and DC load power, respectively. From 0 to 50 s, the demanded power is higher than the PV power. The battery is then in discharging mode. However, when the PV power is higher than the demanded DC power, the battery is charging. The comparison between real data and the digital model data shows a good correlation between them. Indeed, the maximum relative deviations between the real and digital model data are 8% for PV power, 12% for battery power, and 2% for load power.

**Figure 24:**
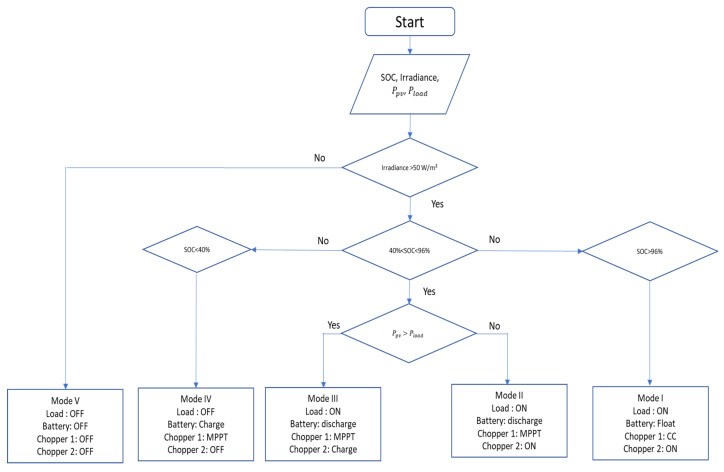
Rule-based algorithm flowchart.

To establish a feedback loop between the virtual and real word of the digital twin, we implement an energy management algorithm in MATLAB Simulink. The rule-based energy management algorithm sends control signals to the PLC (s7 1200). The advantage of using a rule-based control approach is its ease of implementation in real-time [52]. The flowchart of the energy management algorithm is illustrated in Figure 24. It receives measurement data as inputs and generates relay control signals as outputs, which are subsequently transmitted to the PLC.

The energy management system generates five different modes. They are described in Table 7.

**Figure 25:**
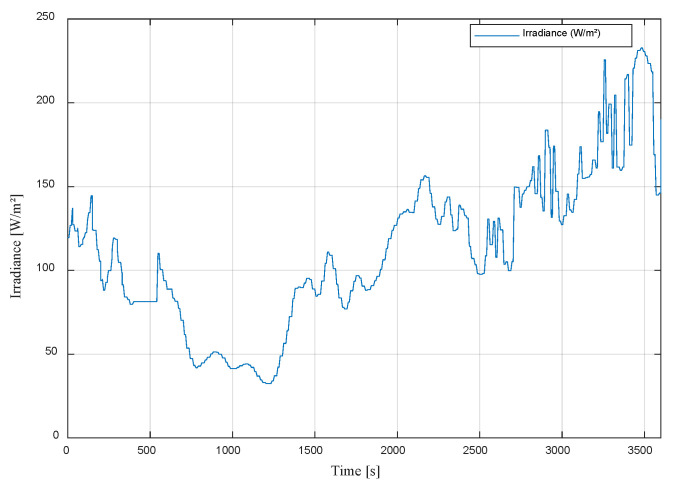
Measured irradiance.

To assess the effectiveness of the energy management system, the initial load is set to 112 W. Figure 25 displays the recorded external solar irradiance for a one-hour period on a cloudy day. Subsequently, these data are transmitted to the dimmer, enabling the generation of adjustable artificial lighting.

**Figure 26:**
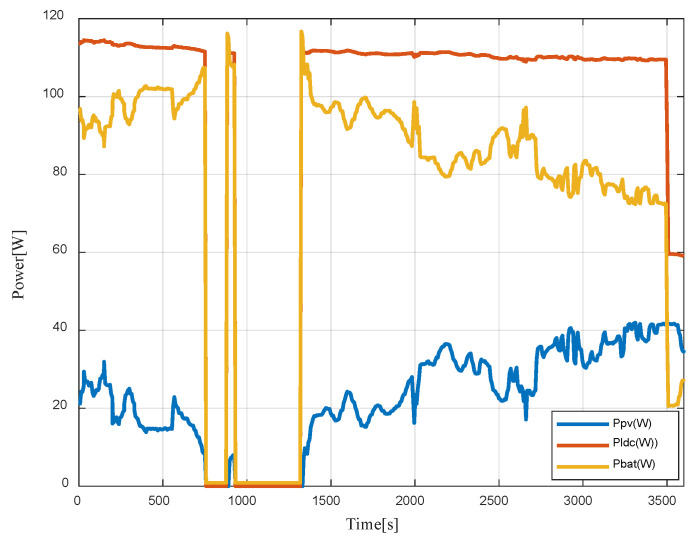
Measured PV, battery, and load power.

Initially, the total power required by the load is provided by both the battery and solar power sources, as the PV alone cannot fully support the load (Figure 26). During the period from 0 to 757 s, the system operates in mode II.

Between 757 s and 876 s, as well as from 932 s to 1317 s, the solar irradiance remains below 50 W/m², leading the system to switch to mode V (system OFF). From 1317 s to 3508 s, the system once again operates in mode II. During the period from 3508 s to 3600 s, the load demand gradually decreases from 111 W to 60 W, causing the battery to generate less power to meet the load requirements.

**Figure 27:**
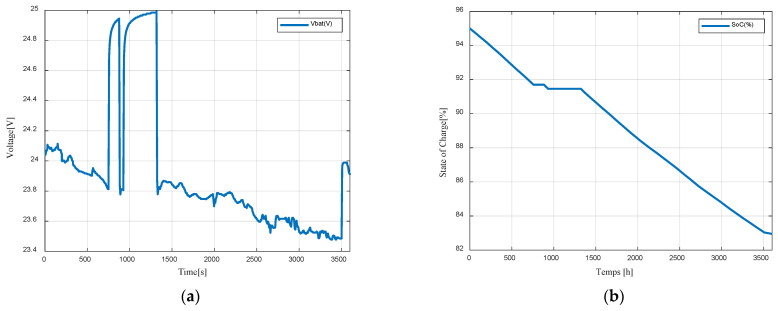
(**a**) Battery voltage; (**b**) Battery state of charge.

Figure 27a shows the battery voltage waveform, while Figure 27b displays the battery State of Charge. The battery primarily operates in discharging mode, except during periods when the system is not in operation (mode I).

## 6. Conclusions

In this work, we discussed the challenges of implementing IoT in renewable energy systems and the potential of digital twins for predicting energy production and consumption. We proposed a framework that combines the use of Internet of Things (IoT) and Digital Twin (DT) technologies for standalone photovoltaic systems. The digital twin is defined as the combination of the physical system and its digital model, allowing for bidirectional data communication. Furthermore, we designed a digital model of the PV system by using Energetic Macroscopic Representation formalism. Experiments performed in real time and their analysis demonstrate the effectiveness of the proposed framework. As future work, we plan to use this platform to explore machine learning’s potential to enhance energy management of PV systems.

## Figures and Tables

**Table 1:** Literature review related to IoT and DT usage for energy management.

Reference	IoT Capability	Multi-Protocols	Energy Management	Monitoring	Digital Model	Digital Shadow	Digital Twin
[34]	✓	✓	✗	✓	✗	✗	✗
[29]	✓	-	✗	✓	✓	✓	✗
[35]	✗	✗	✗	✓	✓	✓	✗
[9]	✓	✓	✓	✓	✗	✗	✗
[36]	✓	✓	✗	✓	✗	✗	✗
[37]	✓	✓	✗	✓	✗	✗	✗
[16]	✗	✓	✗	✓	✗	✗	✗
[30]	✓	-	✗	✓	✓	✓	✗
[38]	✓	✓	✗	✓	✗	✗	✗
[39]	✗	-	✓	✓	✗	✗	✗
[10]	✓	-	✓	✓	-	-	✗
Proposed	✓	Unified environment	✓	✓	✓	✓	✓

**Table 2:** Parameters of the SPR-215-WHT solar panel at STC form datasheet.

Parameter	Value
Maximum power $P_{max}$	215 Wp
Voltage at maximum power point $V_{mp}$	39.8 V
Current at maximum power point $I_{mp}$	5.40 A
Open circuit voltage $V_{oc}$	48.3 V
Short Circuit Current $I_{sc}$	5.80 A
Voltage temperature coefficient $\beta_{oc}$	−136.8 mV/°C
Current temperature coefficient $\alpha_{sc}$	3.5 mA/°C
Number of cells per module $n_{s}$	72

**Table 3:** Parameters of the SPR-215-WHT solar panel at STC form datasheet.

Parameters	Values
$R_{s\_ns}$	0.72262 Ω
$R_{sh\_ns}$	198.7727 Ω
$I_{0}$	$3.8896\times 10^{-15}A$
$A_{ns}$	0.74816

**Table 4:** Parameters of an AGM battery from datasheet.

Capacity	Nominal Voltage	Internal Resistance	Weight
14 Ah/0.7 A	12 V	14 mΩ	4.05 kg

**Table 5:** Some elements of EMR and of control pictograms [48].

Pictrograms	Pictograms Significance
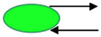	Source of energy
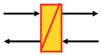	Element with energy accumulation
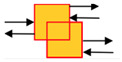	Coupling devices for energy distribution
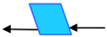	Control bloc without energy accumulation
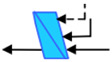	Control block with controller
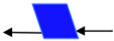	Strategy block

**Table 6:** Direct and indirect inversion of EMR blocks.

	System	Equations	Block Diagrams	EMR and Based Inversed Based Control
Chopper1	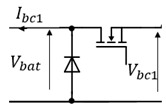	$m_{bc1}=\frac{V_{bat\_meas}}{V_{bc1\_ref}}$ $m_{bc1}=\frac{I_{Lpv}}{I_{bc1}}$	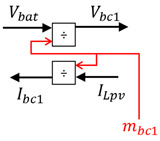	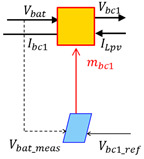
RL filter	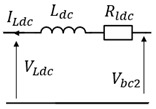	$H\left(s\right)=\frac{\left(\frac{1}{R_{ldc}}\right)}{1+\frac{L_{dc}}{R_{ldc}}s}$	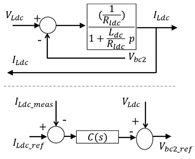	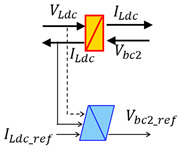
RC filter	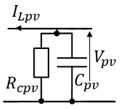	$H\left(s\right)=\frac{R_{cpv}}{1+R_{cpv}C_{pv}s}$	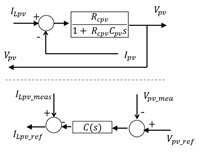	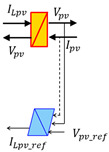

**Table 7:** Different modes of the energy management system.

Modes	Load	Chopper 1	Chopper 2	Battery
**Mode I**: When the battery SOC reaches 96%, which indicates a nearly full charge, the system is operating in float mode. At this point, the PV power output is greater than the load demand. Therefore, the battery should not continue to charge, and the Victron regulator automatically switches to a lower charge voltage mode called float mode to maintain the battery charge level and prevent overcharging.	ON	FLOAT	ON	Float
**Mode II**: This mode is activated when the battery’s SOC is within the normal range of 40 to 96 % and the power generated by the PV panels is lower than the load demand. In such a scenario, the PV panels alone are unable to meet the load requirement, and hence the battery is used to supplement the power supply. The battery operates in the discharge mode, while the PV converter operates in the MPPT mode, and the load remains connected.	ON	MPPT	ON	Discharge
**Mode III**: This mode is activated when the battery’s SOC is within the normal range of 40 to 90 percent and the power generated by the PV panels is higher than the load demand. In such a scenario, the load is powered solely by the PV panels, and any surplus power is utilized to charge the battery. The battery operates in the charging mode, while the PV converter remains in the MPPT mode.	ON	MPPT	ON	Charge
**Mode VI**: This mode is activated when the battery’s SOC drops below 40%, and the power generated by the PV panels is higher than the minimum power required, which is a fixed small value. In such a scenario, the load demand exceeds the PV power output, and the fully discharged battery cannot supplement the power supply. However, the PV panels can still generate power, which can be used to charge the battery after the load is disconnected. In this mode, the PV converter operates in the MPPT mode, and the battery is charging, while the load remains disconnected.	OFF	MPPT	OFF	Charge
**Mode V**: This mode occurs when irradiance is very low or non-existent. In this case, the system goes into complete off mode until solar radiation starts again. The battery charges during the day and is ready to supply the load in case of absence of solar radiation.	OFF	OFF	OFF	OFF
